# Focusing on the value of cooperative learning in physical education: a bibliometric analysis

**DOI:** 10.3389/fpsyg.2023.1300986

**Published:** 2023-11-28

**Authors:** Tong Zhou, Huayi Wang, Dong Li

**Affiliations:** ^1^Department of Physical Education, College of Education, Korea University, Seoul, Republic of Korea; ^2^Department of Education, Kyungil University, Gyeongsan, Republic of Korea; ^3^Department of International Culture Education, Chodang University, Muan-gun, Republic of Korea

**Keywords:** physical education, cooperative learning, teacher training, teaching method, bibliometric analysis

## Abstract

The shift toward cooperative learning has highlighted the growing advantages of individual learning modes during the transition. Nevertheless, a systematic compilation of the precise classification and developmental dynamics of cooperative learning in PE has been absent. This study aimed to organize the existing progress and significance of collaborative learning. The study entailed a meticulous systematic review process, examining 169 articles in this domain with the aid of visualization software. The results of the study indicate that the overall use of cooperative learning in physical education is on the rise and will reach its highest level in 2021; Second, the keywords, major core scholars, journals, countries, and major research topics; the visual knowledge map reveals the major research topics of intrinsic motivation, cooperative learning, motor skills, self-learning, written expression, and pedagogical models. The research primarily centers on primary and secondary education, followed by teacher training and higher education. At the primary and secondary school levels, there is a specific focus on aspects such as motivation, teacher-student relationships, and the group atmosphere. This research also explores sustainable development and training for PE teachers, model integration, and its influence on students’ intrinsic motivation; and finally, the future directions of cooperative learning in PEare summarized. This study provides meaningful and valuable information on how cooperative learning models can be used and developed in various teaching and learning environments, physical education teacher education, and overall student development.

## 1 Introduction

Cooperative learning (CL) has been a topic of interest in the field of education, particularly in physical education (PE) (Dyson and Casey, 2016; Casey and Quennerstedt, 2020; Rivera-Pérez et al., 2021). With the advancement of the Fourth Industrial Revolution (4IR) (Oke and Fernandes, 2020) in the education sector, artificial intelligence presents an undeniable challenge. Along with this, there is a more sophisticated social division of work (Janoski, 2015), team collaboration, and collaborative learning (Peduzzi et al., 2020) in teaching and training, also known as cooperative learning. Stanne et al. (1999) found that cooperative learning is more effective in promoting learners’ movement skill levels (with an effect size of 0.53) than individual effort or competition (with only 0.36) in a meta-analysis of 64 studies. Moreover, PE has been seeking education methods suitable for both social development and student needs in its development over the years. From its development in the 1980s to the present, the 21st-century cooperative learning model has been widely applied and promoted in the education field. It contains five key factors: positive interdependence, individual accountability, group processing, active interaction, and social skills (Johnson and Johnson, 2014). Studies have shown that in PE, CL mainly focuses on the interactions between students and between students and teachers (Metzler, 2017).

CL emphasizes the development of students, including physical, cognitive, social, and emotional aspects, which has been fully reflected in PE. According to Casey and Goodyear’s (2015) systematic analysis based on a qualitative review of 14 articles, quantitative analysis of 11 articles, and mixed-method analysis of 2 articles, CL can promote students’ positivity, listening skills, understanding and encouragement of others, and the ability to build respect, understanding, mutual encouragement, and complete learning tasks together in PE classrooms (Silva et al., 2021a). Most importantly, CL is implemented in a student-centered learning mode, which emphasizes students’ subjectivity (Li and Lam, 2013).

On the other hand, CL in PE can enhance students’ teamwork abilities (Dunn and Wilson, 1991). For example, Sánchez-Hernández et al. (2018) intervened with CL in soccer games, allowing male and female students to participate together by grouping them into teams. This reduced gender discrimination and provided more opportunities for female students to participate. Peer support and collaboration improved physical fitness and reduced gender bias. Furthermore, CL, as an intervention method, can reduce gender differences in the acquisition of motor (Ortíz et al., 2023). The research also reveals that CL can effectively diminish the impacts of gender disparities and inequalities. It promotes the principles and capabilities of sustainable development education among group members, particularly in addressing fairness and inclusivity concerns in early childhood and primary education (Cañabate et al., 2021). In terms of enhancing students’ cognitive and emotional development, CLnot only cultivates students’ level of sports skills but also enables them to identify their weaknesses and learn better by observing others (Barrett, 2005). Through the process of promoting interaction and communication among students, CL also helps to foster good friendships and establish positive relationships among students (Polvi and Telama, 2000).

From the perspective of teachers, CL shifts some of the power from the teacher to the student-centered approach (Silva et al., 2021b). This shift in focus poses a challenge for both pre-service and in-service teachers, breaking the traditional model of PE instruction (Casey et al., 2009). Under this teaching model, there are many uncertain factors and challenges in the implementation of CL (Casey et al., 2015). Therefore, in the process of sustainable professional development for PE teachers, it is important to consider how to implement CL, as well as classroom responses during the implementation process (Casey et al., 2015). For future PE teachers, pre-employment teacher training is needed to enhance the effectiveness of CL implementation (Silva et al., 2021a).

This study offers a comprehensive overview of the development of CL in PE, with a focus on the physical and mental growth of students and the professional competencies of PE teachers. It aims to provide insights into the current research findings and trends in CL through a bibliometric visual analysis, and it is the first bibliometric visual analysis of CL in PE that complements the literature review. By utilizing CiteSpace and VOSviewer visualization software, this research analyzes the knowledge map based on relevant research in the field of CL, to provide some valuable information on the use of CL in physical education and on the professional development of PE teachers.

## 2 Literature review

### 2.1 CL model

CLis a beneficial teaching model that helps to avoid negative competition among individuals. Individualistic learning constantly faces challenges, and therefore, peer-assisted learning and socialization relationships have gradually gained importance in the learning process (Hartup, 1976). In the process of promoting comprehensive student development, physical, cognitive, social, and emotional learning problems become crucial issues that need to be effectively solved in PE (Metzler, 2017). In the teaching and learning process, the aim is to achieve classroom learning goals, and in an ideal classroom, all students can learn how to collaborate with others and how to compete for fun and enjoyment, striving to achieve common learning goals (Johnson et al., 2014). Therefore, in CL, individuals seek to achieve favorable outcomes for themselves or the whole group while improving their own efficiency and that of others, resulting in a situation of mutual assistance and win-win outcomes. This situation is also a way to promote cooperation and create a better future in the 21st century.

### 2.2 Application of CL

The development of PE has often emphasized sports, performance, and achievements, while neglecting other factors and the true needs of students (Bailey et al., 2009). Research shows that CL research is more concentrated in secondary education, for primary and secondary schools to carry out CL in the student motivation, the group is divided into and teacher-student interaction can better promote the learning effect of primary and secondary school students (Bores-Garcia et al., 2021). CL can effectively enhance children’s or adolescents’ participation, socialization with peers, and meet their physical and psychological needs (Jung and Choi, 2016). By encouraging mutual dependence and support among students, they can collectively achieve team honor and academic success (Er and AtaÇ, 2014), leading to improved academic performance (Da Luz, 2015). In such a CL environment, a safe and motivating atmosphere can be created for students. Particularly in PE, the CL is especially important as it helps students avoid being passive observers, which can often occur in PE classes divided into practice and theory units.

Currently, CL has a positive impact on students’ behavioral motivation, from elementary to high school and university sports cooperation courses, and affects students’ cognitive, physical, and emotional performance (Liu and Lipowski, 2021; Rivera-Pérez et al., 2021). In addition, using CL can improve teachers’ satisfaction with the classroom (Gil-Arias et al., 2022), enhance teaching quality, evaluation (Lopez et al., 2008), and promote curriculum reform and optimization under the background of technology and information (Fernández Río, 2017; Kang and Kang, 2019), the multimodal approaches to math and PE within CL is beneficial in fostering the academic and social attitudes of pre-service teachers (Bassachs et al., 2022), and promote sustainable professional development of PE teachers (Goodyear, 2017). Especially with the development of intelligent systems (Yoda, 2017), networks, and modern scientific and technological advances (Jastrow et al., 2022), CL can be combined ingeniously, adapting to different situations and promoting the comprehensive development of students.

### 2.3 Value of bibliometrics

The application of bibliometrics in scientific research has been widely recognized, as it provides a useful tool for scholars to gain insights into current research topics and expand their knowledge base (Hood and Wilson, 2001). With the ability to quickly sift through and cluster large volumes of literature, bibliometric tools such as CiteSpace and VOSviewer not only accelerate the production and dissemination of information, but also improve the utilization and visualization of information (Chen et al., 2017). Bibliometric analysis can help researchers by providing a quantitative approach and can review the existing literature in a particular field, helping researchers to systematically understand the current state of the field and future trends (Zhou et al., 2023a). Bibliometrics is widely used in independent fields and also in cross-disciplinary fields, contributing to the development of multidisciplinary intersections (Song and Wang, 2020). In addition, based on the development of modern computer technology, the results of this research field are presented visually with graphical and visual results that can further complement the literature analysis (Donthu et al., 2021). Bibliometrics can be valuable for educational purposes as it offers learners intuitive and visual information about research in a specific field. It assists learners in locating key literature more efficiently and comprehending important information about a particular field or themes (Huang et al., 2020). Additionally, bibliometrics can enhance the quality and speed of scientific research.

Additionally, these tools, in combination with computer program processing capabilities, can assist scholars in organizing, summarizing, and analyzing specific research topics. Through knowledge maps, detailed information about publications, keywords, authors, countries, institutions, journals, and even co-citation relationships can be intuitively presented. As of March 2023, bibliometrics has been widely applied in fields such as environment (Mao et al., 2018), chemistry (Shen et al., 2022), education and intelligent learning (Chen et al., 2021), mental health (Akintunde et al., 2021), and sports medicine (Chen and Shin, 2021). However, there has been limited application of bibliometrics in sports education, and no scholars have conducted a visual analysis of CL.

Therefore, this study aims to conduct a bibliometric analysis of the development of cooperative learning in sports education. The objectives of this study are:

1) To determine the extent of publications, primary literature, research topics, keywords, authors, and journals in the field.
2) To identify collaborative networks and partnerships among authors and countries.
3) To analyze the information presented in the visual knowledge map.
4) To comprehend the progress of CL in PE and offer future prospects.

## 3 Method

### 3.1 Database search

**Figure 1:**
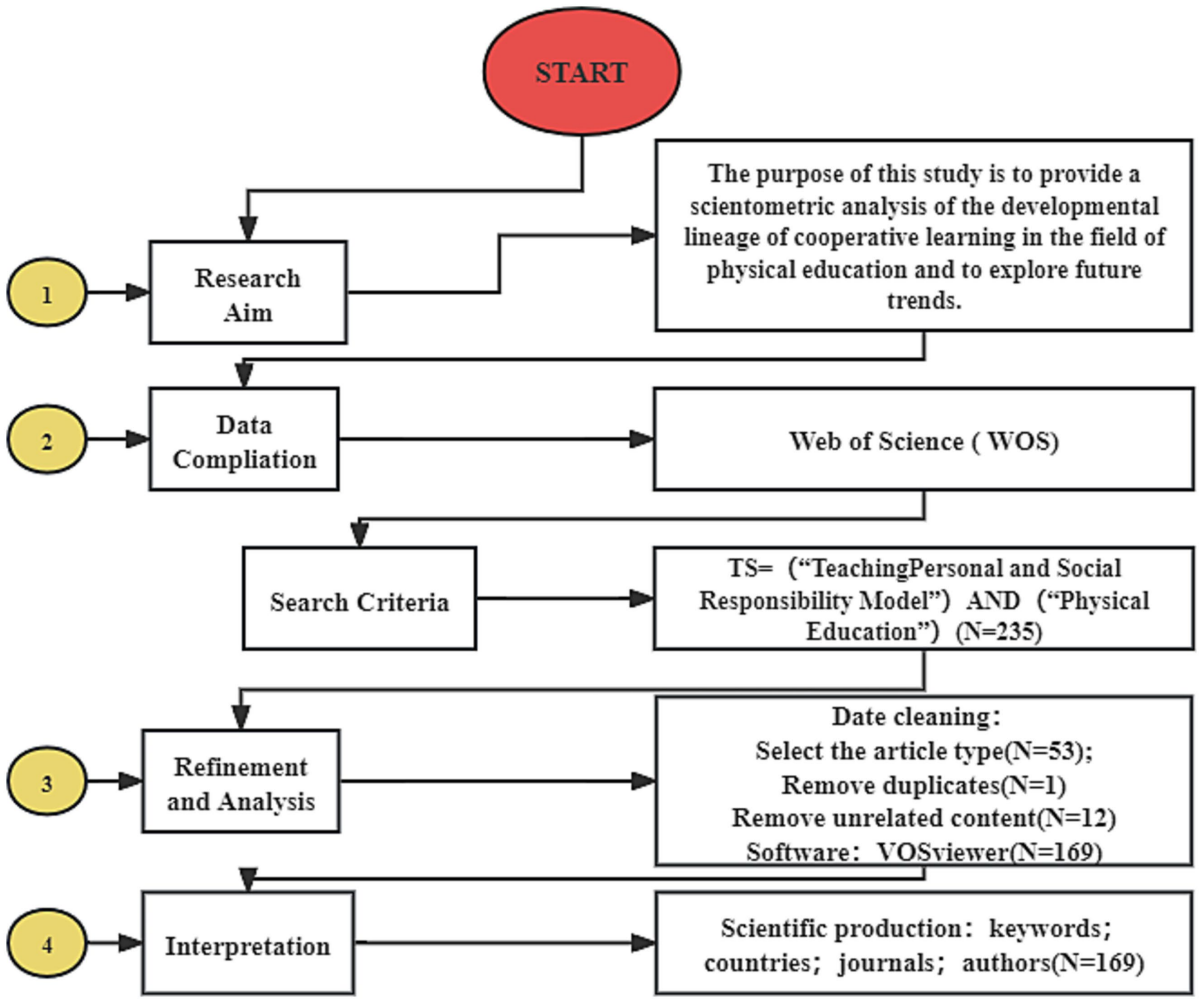
Main steps of the study process. Adapted from Leal et al. (2022).

This section consists of three main steps, namely establishing a database of total records, analyzing the organized data using visualization software to create a knowledge map and evolution analysis. Following the PRISMA (Preferred Reporting Items for Systematic Summaries and Meta-Analyses) flow chart paradigm for literature filtering, the next step was data cleaning (Page et al., 2021). To achieve this goal, four main steps were followed (Figure 1).

The first step involved clarifying the research purpose, which is to introduce the research achievements of cooperative learning in the field of physical education using bibliometric analysis.

The second step involved selecting the literature database. Web of Science (WOS) Core Collection was chosen as the main literature retrieval database for bibliometric analysis due to its high-quality research data, large audience, and long history (Mongeon and Paul-Hus, 2016). The advanced search function of WOSCC was used to conduct a search with the following keywords: TS = ((“Cooperative Learning” AND “Physical education”) OR (“Collaborative Learning” AND “Physical Education”)) (Bores-Garcia et al., 2021). To avoid omissions, a time span was not selected, and instead, the time period was set to end in 2023. The complete records and all relevant information related to the articles cited in the database were then exported. CiteSpace and VOSviewer were used as visualization software to conduct bibliometric analysis. These two software packages can complement each other, thus improving the quality of the visualization knowledge map (Zhou, 2023).

The third step involved data cleaning of the obtained literature. Only articles were selected, duplicates were removed, and withdrawn papers were excluded. Additionally, papers that contained irrelevant titles, abstracts, and keywords were also removed. Based on previous studies, VOSviewer version 1.6.16 was used to generate the results. After the final filtering and screening, a total of 167 paper documents related to cooperative learning in physical education were included in the WOS Core Collection database. These papers were published in 69 publications, originating from 36 countries and 185 institutions, and authored by 380 authors. The literature sources were mainly from education, sports, and information technology.

### 3.2 Data analysis

Bibliometric analysis covers many factors such as structural presentation, dynamic changes, evaluation, prediction, and scientific measurement in the current field, and the methods adopted are more systematic. In addition, both CiteSpace (Chen, 2006) and VOSviewer (Van Eck and Waltman, 2010) were used in the third step as commonly used software tools for creating literature networks (such as keyword cluster analysis, author analysis, and country analysis) and shared citation information among literature (such as shared cited authors and shared cited literature).

Finally, all the results, including keywords, authors, countries, and publications, were presented in tables and visualization knowledge maps through data analysis.

## 4 Results

### 4.1 Dynamic trends in CL

**Figure 2:**
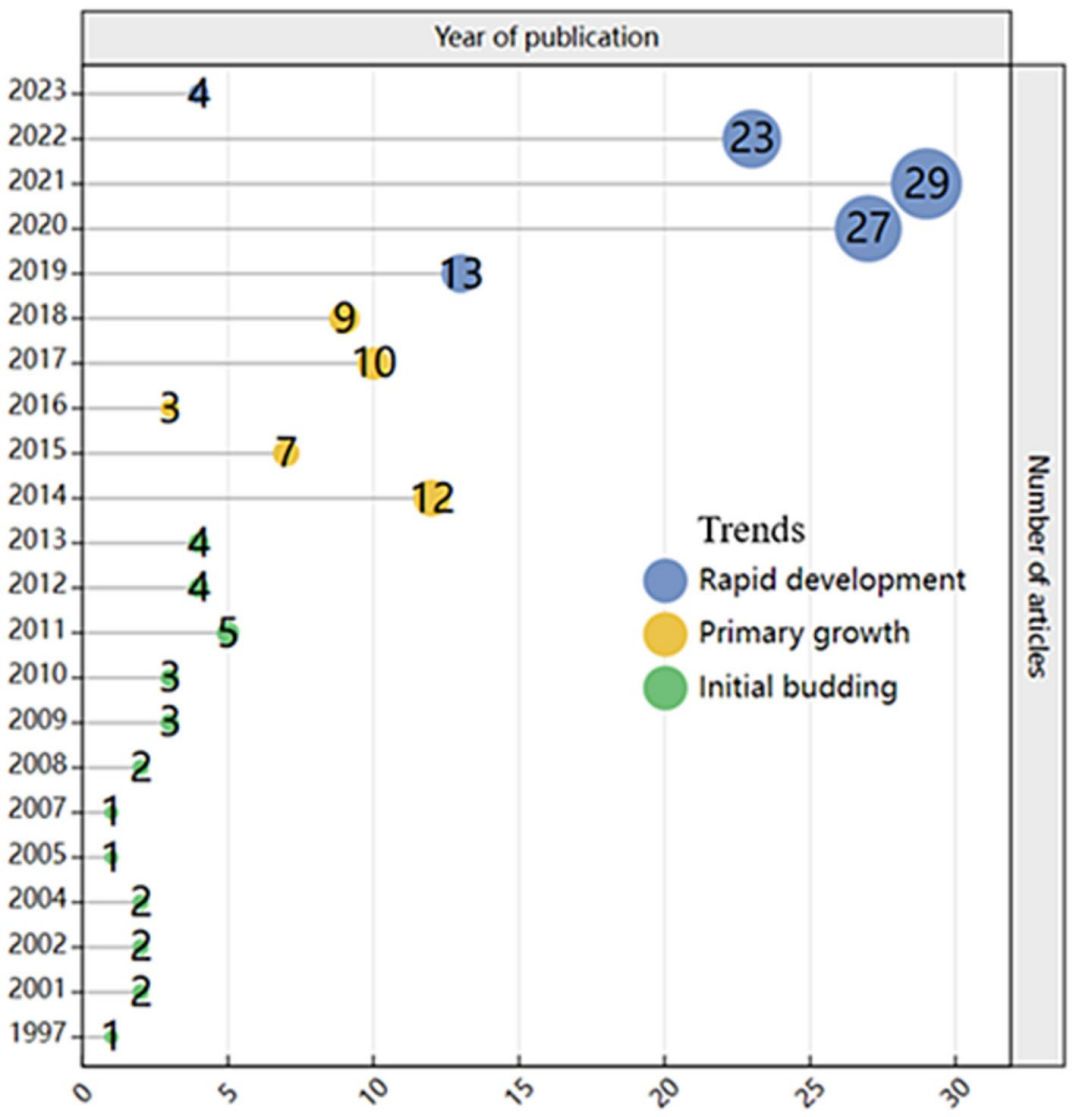
Trends in annual publication volume of CL in the field of PE.

From Figure 2 it can be seen that CL has been applied in the field of education for a long time, but it was not until 1997 that it began to be used inPE. The previous literature search found that it was about social and moral education for high-risk children, that is, moral education in the process of human development (Miller et al., 1997), and it was an empirical study. From 1997 to 2023, the trend showed a slow increase to rapid growth. Spain has the highest number of publications on cooperative learning in physical education, with 71 papers cited up to 404 times, indicating a significant amount of research in this area. The second highest is the UK, with 21 papers published and cited up to 1,842 times, mostly empirical studies that explore current teaching methods. The USA has published 20 papers and cited up to 928 times, also mostly empirical studies. China and France had 13 and 8 publications, respectively (Table 1).

**Table 1:** Status of publication volume in the 10 most relevant countries.

Rank	Country	Documents	Citations	Total link strength
1	Spain	71	404	5
2	England	21	1,842	12
3	USA	20	928	8
4	People’s R China	13	23	1
5	France	8	124	0
6	Ireland	7	462	7
7	New Zealand	6	222	6
8	Sweden	6	98	2
9	Turkey	6	52	0
10	Norway	5	20	1

**Figure 3:**
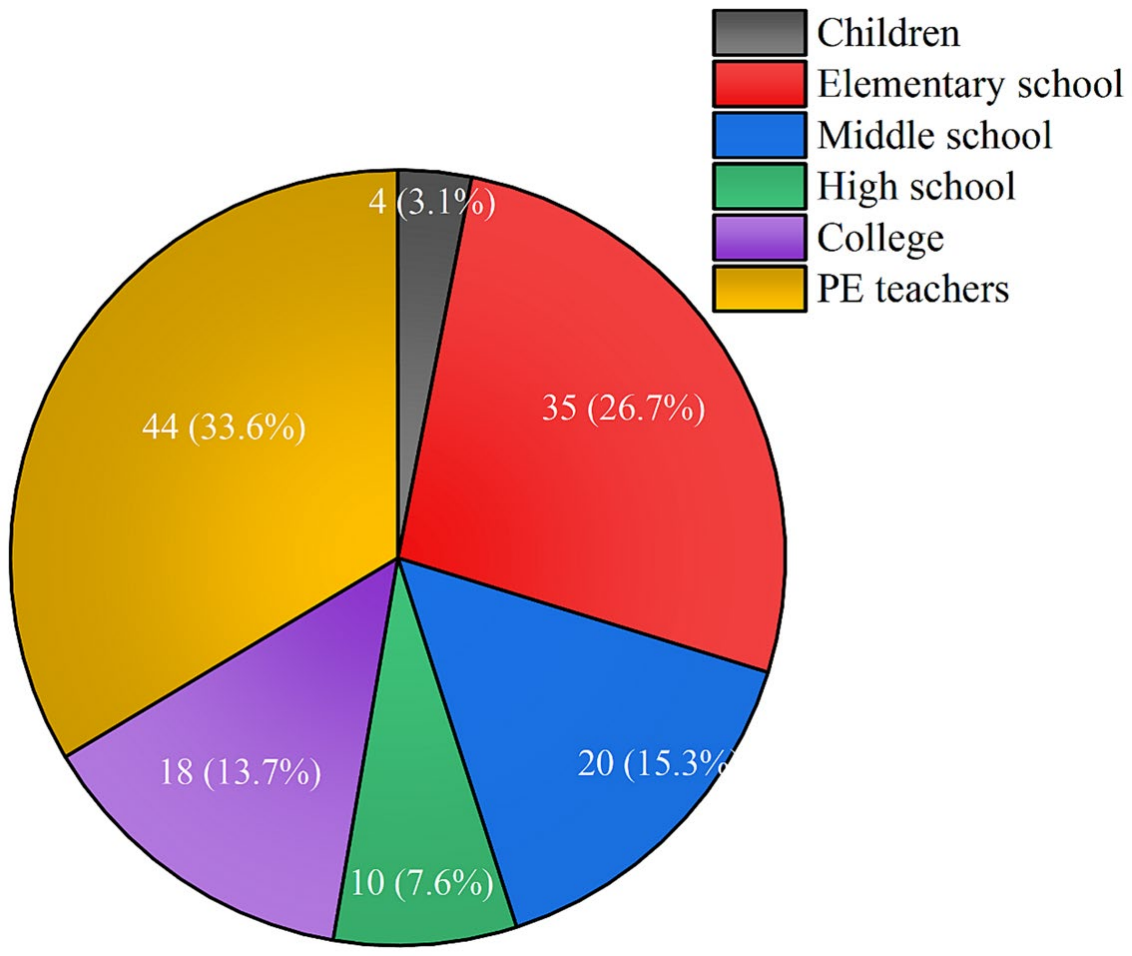
Research levels of CL in PE.

In the research findings, it was observed that among the literature primarily centered on students, studies utilizing Collaborative Learning (CL) were conducted with children (*N* = 4, 3.1%), at the elementary school level (*N* = 35, 26.7%), middle school level (*N* = 20, 15.3%), high school level (*N* = 10, 7.6%), and university level (*N* = 18, 13.7%). Literature primarily focused on teachers as the subjects of research accounted for 44 articles, representing 33.6% of the total. In terms of the application of CL in PE, there is a greater concentration of research aimed at primary and secondary education, as well as teacher-centered studies (Figure 3).

### 4.2 Keyword knowledge graph analysis

**Figure 4:**
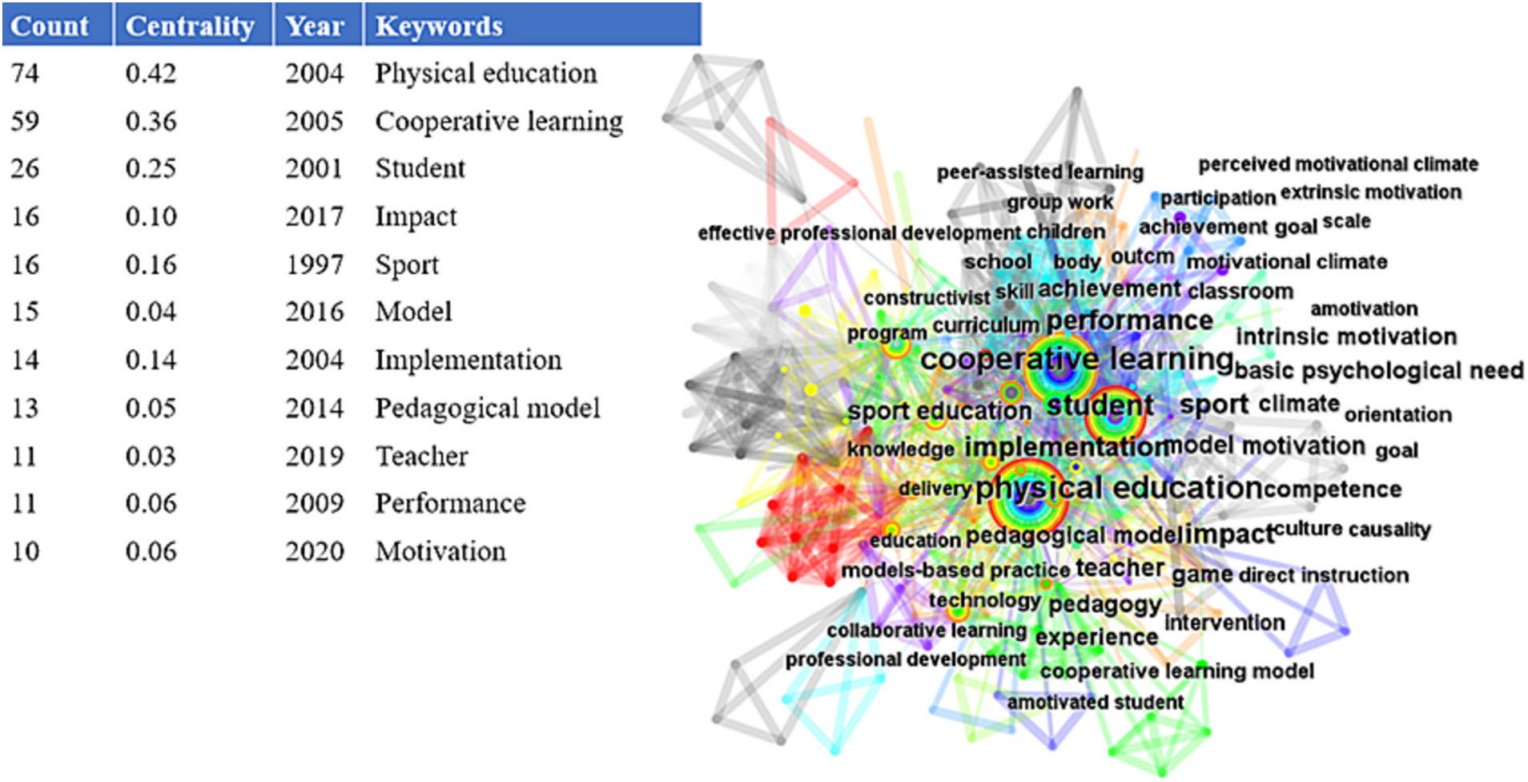
Keyword visualization analysis.

Using CiteSpace to perform cluster analysis and centrality calculation of keywords in the current field (Figure 4), we can see that the clustering is mainly cantered around “physical education” and “cooperative learning,” The size of the circles represents the frequency of appearance of the keywords, and the outermost circle color represents frequent appearance in recent years. In terms of the application of CL in PE, it is mostly carried out through the relationship between teachers and students. Therefore, the centrality of “teacher” and “student” as keywords reaches 0.03 and 0.25, respectively, and the centrality of “student” is 0.22 higher than that of “teacher,” which is consistent with the goal of CL mainly targeting students for teaching (Li and Lam, 2013). Moreover, as a model, CLis also constantly exploring the factors that may affect it. Therefore, the centrality of “model” and “educational model” as keywords is 0.04 and 0.05, respectively.

**Figure 5:**
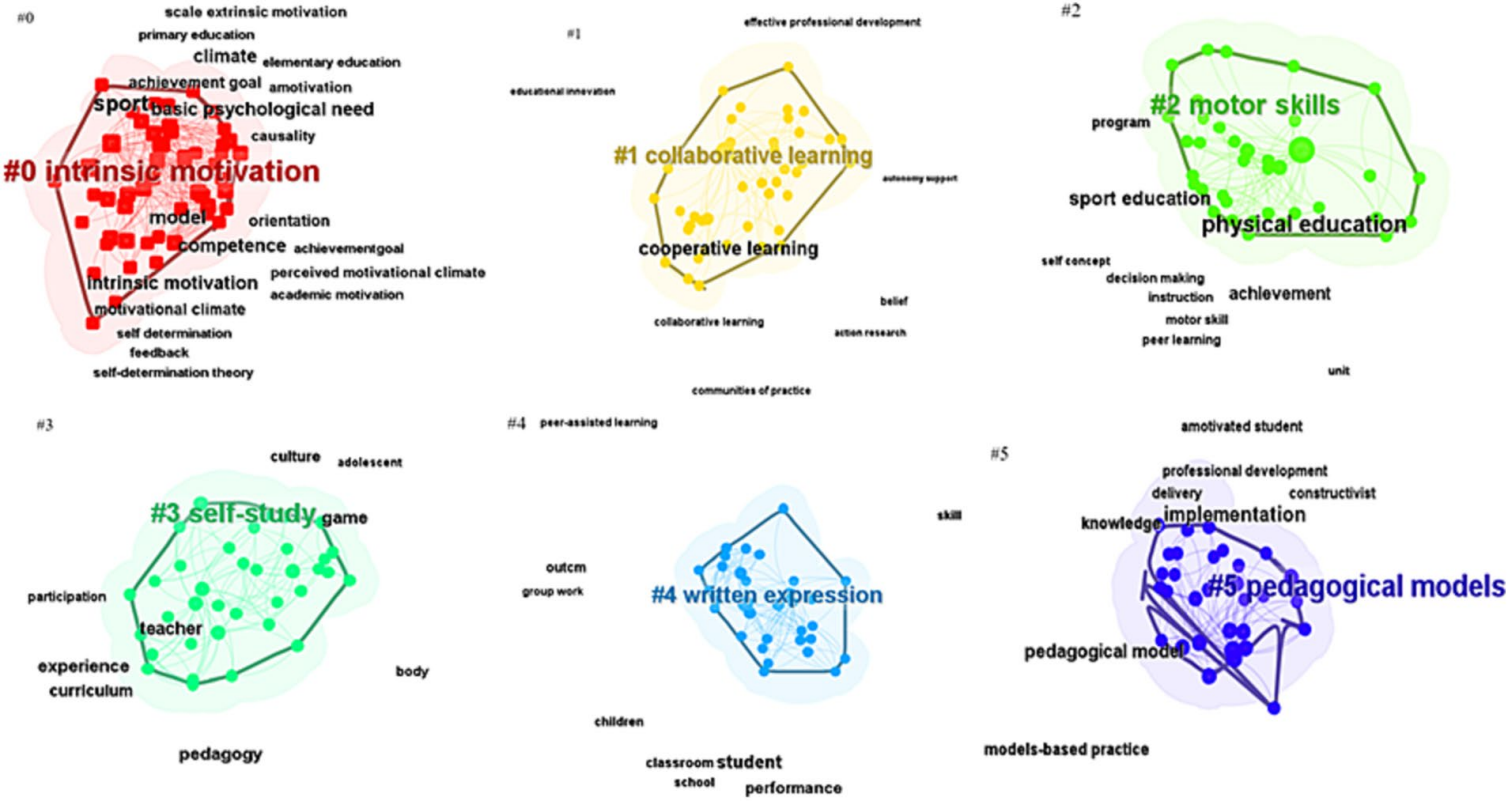
Keyword clustering knowledge graph.

**Figure 6:**
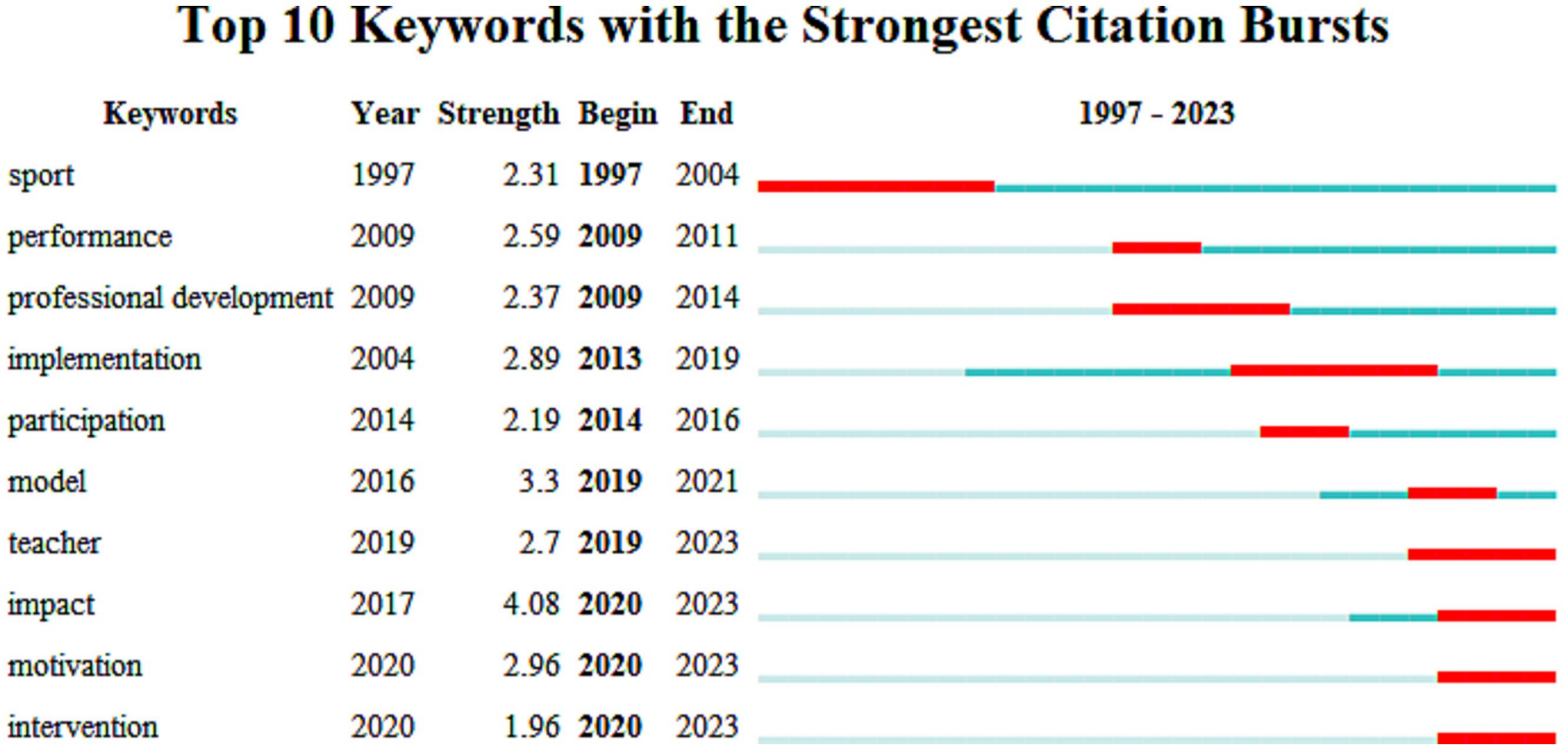
Visual analysis of key outbreak words.

In Figure 5, six cluster themes related to research are identified, which include intrinsic motivation, cooperative learning, motor skills, self-learning, written expression, and educational models. The explosive period of keywords in the application of CL in PE was visually analyzed, and it was found that the keyword “implementation” had a relatively long duration from 2013 to 2019, lasting for 6 years, which was a breakthrough in empirical research on the application of CL in the 21st century. Its impact strength value was 4.08, and it has had a significant amount of research on the roles and influences of students and teachers, which has continued from 2020 to 2023. “Motivation” had a strength value of 2.96 and has positive implications for intrinsic motivation, behavioral motivation, and student behavior in the classroom. “Teacher” had a strength value of 2.7, and “intervention” had a strength value of 1.96. These four main keywords have continued research possibilities in 2023, and they are related to COVID-19, different PE teaching modes (Colao et al., 2020), online CL (Ivone et al., 2020), blended education (Hamzah et al., 2022), etc. in Figure 6. The use of CL in different scenarios is a challenge and discovery that needs to be continuously explored in the future.

### 4.3 Author knowledge graph analysis

Table 2 provides an analysis of the authors, focusing on the top five authors in terms of publication volume and relevance to the main topics. It was found that three of the authors are affiliated with institutions in Spain, with publication volumes of 19, 14, and 5, and average citation rates of 11.0, 68.9, and 5.0, respectively. Spanish scholars are leading the way in research related to CL inPE, with Goodyear, Victoria A. having published 7 articles with an average citation rate of 74.1. Dyson, Ben from the United States has published 5 articles with an average citation rate of 21.6. This is consistent with the country ranking in Table 1, where Spanish scholars account for about half of the total publications. Spanish scholars have conducted a lot of empirical research in this field. It was found that their research has a significant impact on students’ classroom satisfaction with teacher CL training and attitudes (Martínez Benito and Sánchez Sánchez, 2020). They have also explored to varying degrees the integration of CL with other sports models, such as PE and game-based learning models (Chiva-Bartoll et al., 2018; Evangelio et al., 2021).

**Table 2:** Analysis of the top 5 authors by number of published articles.

Rank	Country	Author	Documents	Citations	Average citations
1	Spain	Fernandez Rio, Javier	19	210	11.0
2	Spain	Casey, Ashley	14	965	68.9
3	England	Goodyear, Victoria A	7	519	74.1
4	Spain	Rivera Perez, Sergio	5	25	5.0
5	USA	Dyson, ben	5	108	21.6

### 4.4 Visualization analysis of literature and journals

**Figure 7:**
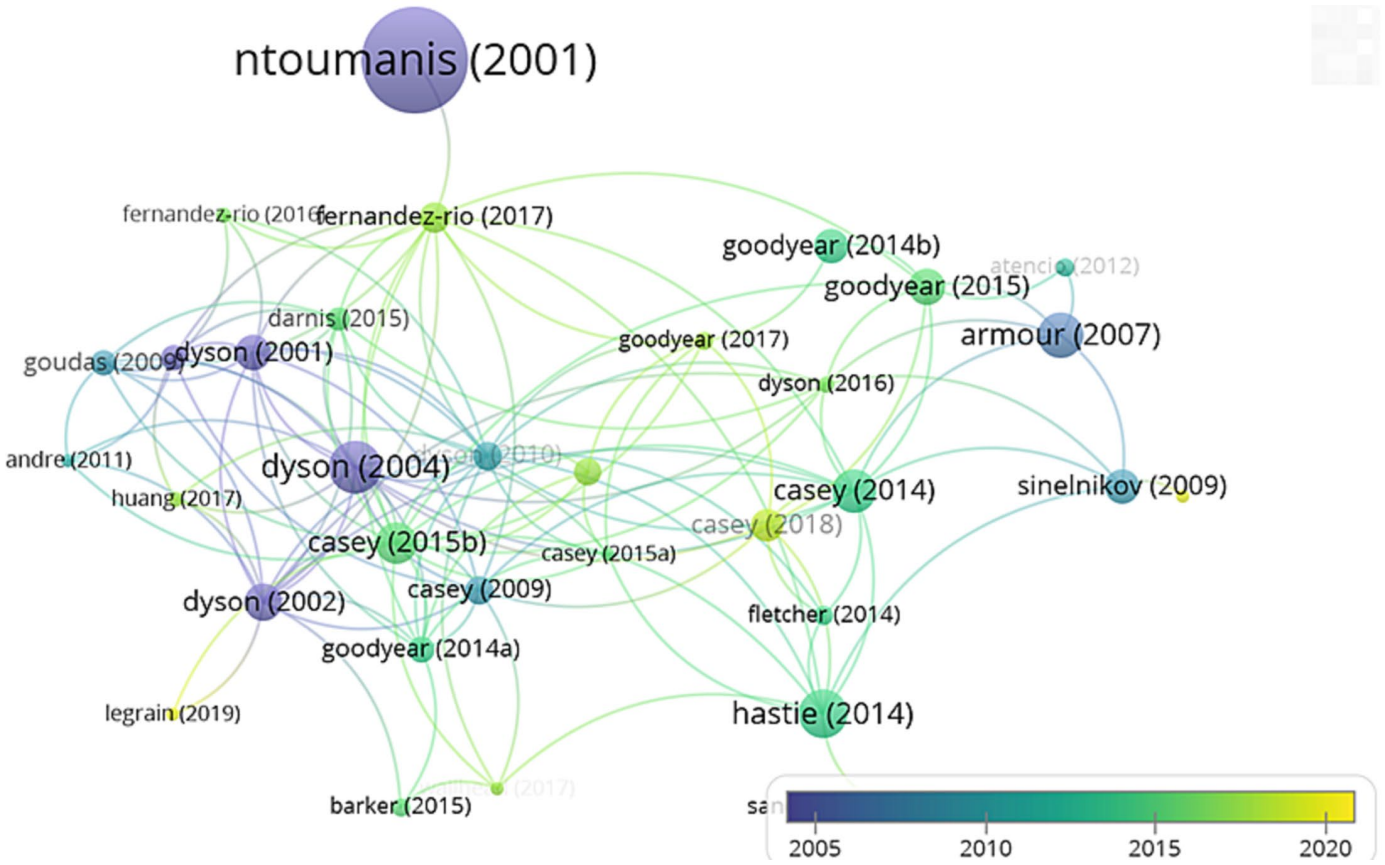
Visual knowledge map of the main literature.

Through the visualization analysis of the knowledge graph of literature (Figure 7), the size of the circle represents the frequency of citation of the literature, and the color change indicates the publication year. Ntoumanis (2001) has the highest citation frequency for a single paper, which empirically analyzes the perception of movement ability and the mobilization of students’ intrinsic motivation, emphasizing their importance in PE classes. According to Dyson et al. (2004), who are the second most cited literature, they have provided a deeper understanding of the model of PE. They emphasize that teaching should be student-centered in various PE scenarios, rather than being focused on the teacher. This is also a focus of CL, to empower students to take the lead and improve their self-directed learning abilities while engaged in cooperative learning.

**Figure 8:**
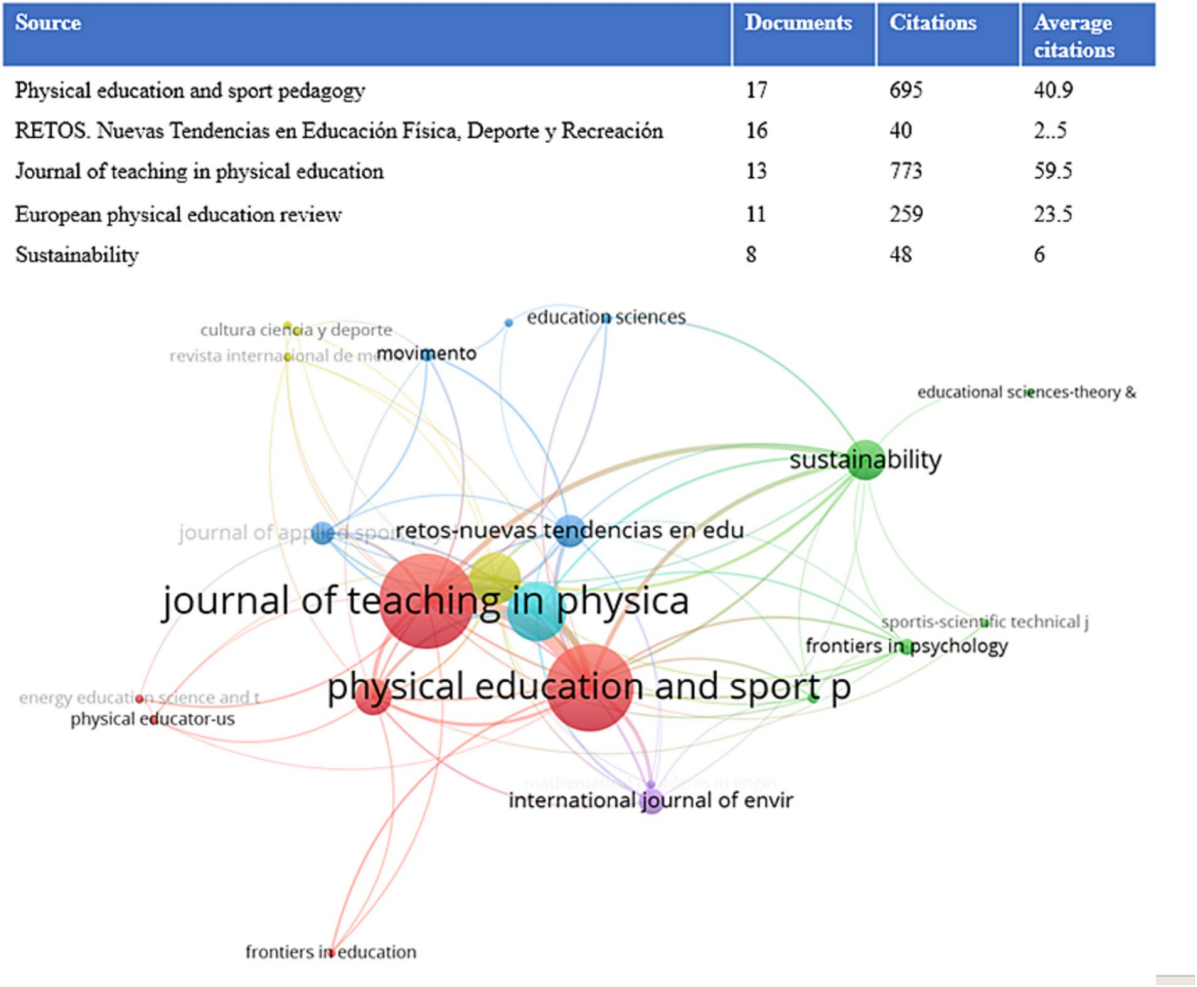
Visual knowledge map of major journals.

As shown in Figure 8, the number of articles on CL in PE is mainly concentrated in *Physical Education and Sport Pedagogy*, with 17 articles published, cited 695 times, and an average citation frequency of 40.9 times, indicating the high quality of the articles. The analysis found that all of them were empirical analyzes, which studied the positive effects of CL on enhancing students’ social skills (Silva et al., 2021a), improving their participation in class, and promoting the formation of lifelong values while enhancing their sense of personal responsibility (Luo et al., 2020). Moreover, it has a positive impact on the use of teaching methods by PE teachers and the sustainable development of their profession (Guzmán and Payá, 2020).

**Figure 9:**
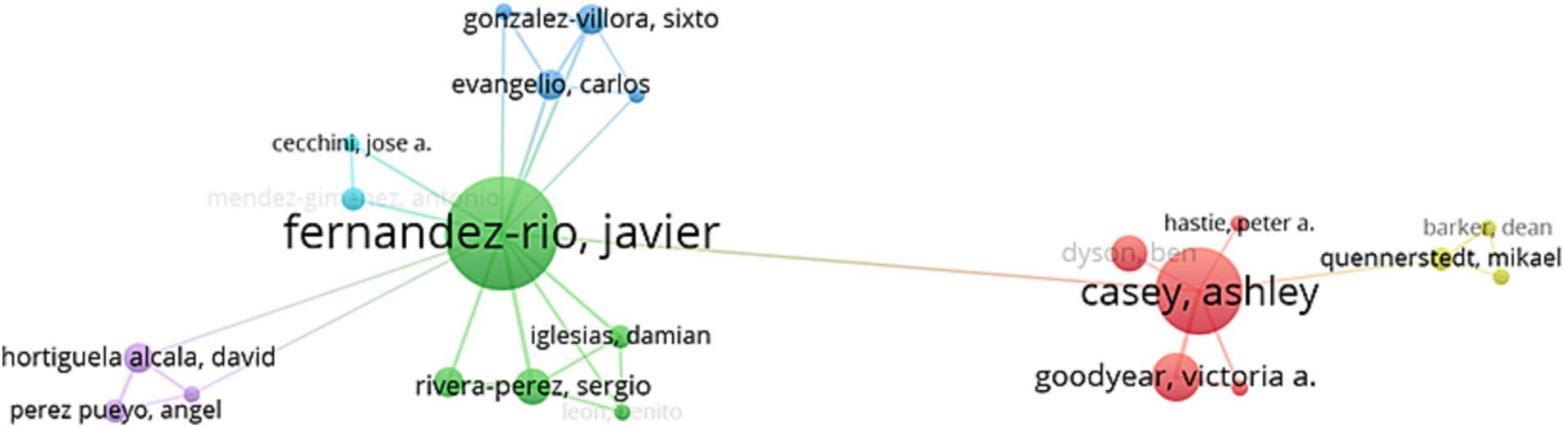
Author collaboration visual knowledge graph analysis.

Figure 9 shows that the *Journal of Teaching in Physical Education* is the most cited journal source, with a citation frequency of 454 and a connection strength of 8,673. *Physical Education and Sport Pedagogy* is the second most cited, with a frequency of 264 and a connection strength of 6,097. This is followed by *Sport, Education and Society*, *Quest*, *and European Physical Education*, with citation frequencies of 187, 147, and 143, and connection strengths of 4,192, 3,598, and 3,389, respectively. The connection strength can help analyze the degree of closeness of the journal to this topic.

### 4.5 Visualization analysis of author and country cooperation

**Figure 10:**
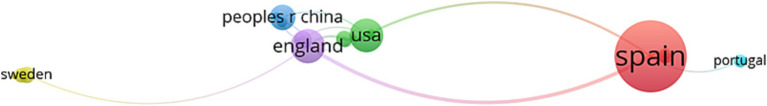
Visual knowledge map of country cooperation.

A visualization analysis was conducted on authors who have published 2 or more articles together, as shown in Figure 10. It can be observed that there are roughly 4 clusters with 5 different colors, and the thickness of the lines represents the strength of the connection between authors. The larger the circle, the stronger the author’s core leadership ability in collaborative research, and the collaboration is mainly led by scholars from Spain.

Visual analysis of collaboration between countries reveals that there is no clear clustering due to lack of collaboration between countries. The top-ranked country in terms of publication output, Spain, has closer collaboration with Portugal, the United Kingdom, and the United States, but collaborations with other countries in this field are relatively scarce. Therefore, future research should expand the scope of collaboration among countries.

### 4.6 Timeline graph

**Figure 11:**
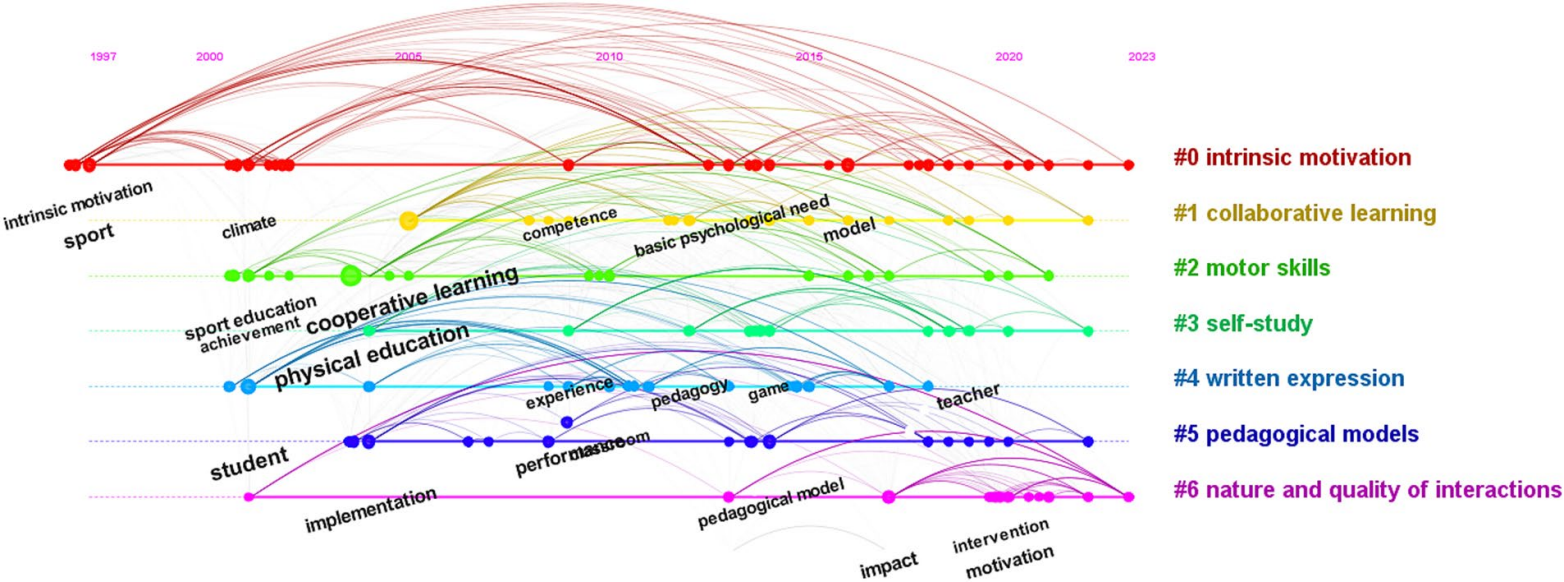
Timeline of keywords for CL in PE.

In visualization software, a timeline chart (Figure 11) provides a clearer and more concise understanding of the main research topics in a specific field during a certain time period. Cluster 0 includes key themes such as intrinsic motivation, sports, action, and self-directed learning, which have been continuously researched from 1997 to the present. In sports teaching, student motivation, whether intrinsic or extrinsic (Fernandez-Rio et al., 2017), is influenced by factors such as teachers, methods, and models. Cluster 1 mainly focuses on cooperative learning models (CL), Teaching Games for Understanding (TGfU) (Gil-Arias et al., 2020), Jigsaw and Team Games Tournament (TGT) (Garcia, 2021), PE models, and the development of theories in mixed or individual models. Cluster 2 is mainly about sports skills, PE, achievement, and peer-assisted learning. CL has been found to have a positive impact on students’ skill development and correction, academic achievement (Ghaith, 2002), and learning in different projects. Cluster 3 is about self-directed learning, which is related to the individual development of students in CL. In order to help the team achieve better results, students will make their best efforts to complete their assigned tasks, ultimately resulting in better team performance. Cluster 5 is similar to Cluster 2, both exploring teaching models in the field. Cluster 6 mainly focuses on the positive effects of CL classrooms on students after empirical interventions. The socialization of students, communication and cooperation between individuals, and the promotion of personal responsibility all contribute to the development of their moral level (Yunanda et al., 2018). In addition, new keywords that emerge from the combination of CL with digital information technology and artificial intelligence are worth exploring. In summary, these major clusters are all based on the expansion and exploration of “cooperative learning” and “physical education.”

## 5 Discussion

The purpose of this study is to use bibliometric methods to analyze the knowledge structure of CL in the field of PE. Through bibliometric analysis, a total of 167 literature references were identified in this field, coming from 69 publications, 185 institutions in 36 countries, and 380 authors. Bibliometrics, as a quantitative tool and analysis method, greatly reduces the subjectivity in information indexing and retrieval, especially when combined with visualization analysis. This can facilitate the identification of potential collaborators for scholars or countries in the field of CL. The application of CL in PE has evolved from simply adapting models to diverse development, demonstrating the superiority of CL in PE.

### 5.1 Dynamic trends in CL

According to the results analysis, there are four main parts. Firstly, in the field, the first three periods (1997–2013) were in the initial growth stage, during which CL was just beginning to gain attention. From 2014 to 2018, CL showed significant growth in PE, attracting scholars’ attention and being mainly empirical research to verify the learning environment for students (Chiva-Bartoll et al., 2018) and explore the impact of CL on PE (Huang et al., 2017; Sánchez-Hernández et al., 2018). The reason for this growth is mainly related to the popularity of social media and online collaboration tools, which enable multiple people to collaborate on tasks and facilitate interest and research in CL. In 2016, there were only three papers published, which may not be the peak period for CL research in PE, leading to relatively few researchers publishing relevant papers.

From 2019 to 2023, there is a rapid growth period mainly related to online education and distance learning. With the outbreak of COVID-19, many schools or institutions were forced to switch to online education, promoting research and exploration of online learning and cooperation (Yoo et al., 2021). At the same time, the development and breakthrough of emerging scientific technologies and new methods and tools continue to emerge, stimulating more research interests. For example, exploring teaching using different factors and combining computer information and communication technology such as artificial intelligence (Zhou et al., 2023b).

### 5.2 Information on the value of CL

Perform visual clustering on the second set of keywords, focusing on six clustered topics related to intrinsic motivation, cooperative learning, motor, self-directed learning, influence, and educational models.

In PE teaching environment, students’ intrinsic motivation is influenced more by teachers and peers. CL can help students improve their learning enthusiasm, establish a good sports atmosphere, and actively participate in activities (Liu and Lipowski, 2021). CL is also key to building good friendships among peers. Facing current issues such as depression and social isolation among students, strengthening communication and cooperation among students is crucial (Polvi and Telama, 2000). A study investigating the impact of an ongoing CL intervention on student motivation has found that CL can enhance students’ intrinsic motivation. Additionally, it also confirmed that students hold positive perceptions of the classroom atmosphere created by CL (Fernandez-Rio et al., 2017).

From the beginning, CL was intended to promote students’ learning of motor (Altınkök, 2017), and to promote students’ physical and cognitive development. In the use of CL for teaching basketball classes, it was observed that students’ basketball skills improved, and students were able to establish positive interpersonal relationships. This study emphasizes the importance of employing suitable grouping methods based on the teaching objectives (Yang et al., 2021). Through peer collaboration, mutual supervision, correction, and learning, cognitive development has also been enhanced, and the keywords are mostly centered around this topic.

Regarding self-directed learning, students are grouped in CL, with each person having different tasks. To complete the tasks, each person needs to independently complete a portion of the task (Janoski, 2015). Through a controlled group experiment involving 96 students in a handball teaching unit, it was confirmed that the mixed model of CL and TGfU significantly impacts the motivational atmosphere among students compared to traditional teaching methods (Chiva-Bartoll et al., 2018). There were also notable differences in students’ autonomous participation. This promotes the development of self-directed learning, focusing on influencing self-development, independent learning, and other related topics.

The exploration and influence of teaching models are also mostly explored through empirical research, with the integration and exploration of different PE models such as physical education model, teaching games of understanding model, and teaching personal and social responsibility model all recognized through teaching experiments (Kirk, 2013; Kipp and Bolter, 2020; Anggreni et al., 2022). In the blend of the teaching models for PE and CL, teachers found that after 24 sessions of continuous implementation, they fostered a conducive learning environment, leading to comprehensive student development across various domains (Gil-Arias et al., 2022). However, in the application of the model, it is emphasized that to ensure the authenticity and effectiveness of data within the implementation of CL, it is essential to integrate CL scenarios to secure the authenticity of the implementation (Casey et al., 2015). In addition, it is clear from the keyword explosion chart and the timeline of keywords that current research is mainly focused on teachers, motivation, influence, as well as current digital communication and artificial intelligence.

Specifically, the research findings indicate that CL is more commonly employed in education focused on elementary and middle school levels with students as the subjects of study. Notably, at the elementary and middle school levels, there is a stronger emphasis on student motivation, creating a motivating atmosphere, and promoting teacher-student interactions, consistent with previous research findings (Bores-Garcia et al., 2021). Approximately 44 studies (33.6%) primarily center around teachers as the subjects of research. In research centered around teachers, the primary focus lies on assessing teachers’ pedagogical competence and the utilization of instructional methods. Through a study of multiple cases, it has been observed that the implementation of CL in diverse cultural settings can result in disparate effects, posing a challenge for educators in terms of instructional strategies (Karmina et al., 2021). Hence, to better facilitate the training of PE teachers in implementing CL within intricate settings, it is imperative to address challenges that may emerge during the process. These challenges encompass effective time management, fostering student motivation, and ensuring active participation in physical activities within designated areas. Additionally, research emphasizes the necessity of a strong integration of collaboration within the sports domain (Hortigüela-Alcalá et al., 2020). In addition, studies involving college students primarily concentrate on whether CL can effectively improve learning outcomes, foster students’ intrinsic motivation, increase their level of engagement, and enhance satisfaction. One study has indicated that while CL is successful in boosting social and interpersonal skills, it may not be the most efficient method for enhancing learning outcomes, and it does not show any significant gender differences (Baena-Morales et al., 2020). However, CL does appear to encourage greater participation of female students in sports activities (Goodyear et al., 2014).

On the other hand, CL has shown significant benefits for the psychological and communication development of children (*N* = 4, 3.1%). CL can effectively improve children’s negative emotions (Jiao et al., 2021). Specifically, CL can aid children, including those with autism, in increasing their interaction frequency with peers in physical education and free play environments, reducing instances of inappropriate interactions (Lee et al., 2022).

In the context of authorship in Spain, visual information analysis shows that Spanish scholars have conducted research mainly on the evaluation of teachers’ activities in CL in PE (Bermejo et al., 2020) conducted an empirical study on students’ satisfaction, performance, participation, and teacher evaluation diaries in the classroom through CL and found that students submitted very satisfied scores, with scores above 8 out of 10. This also proves that CL as a teaching model should not only be a method. Spain has small-scale contacts with countries such as the United Kingdom, the United States, and China, and in the future, it is necessary to continue to expand contacts between countries and scholars worldwide. Through literature analysis, American scholars have conducted empirical research on PE classes in primary and secondary schools, while China has continued research on PE classes for college students. The United Kingdom has examined CL, aiming to understand the potential and challenges of CL, improve the sustainable development of teachers’ professions, avoid structural constraints on teaching, and actively participate in CL cases. The adaptability of CL in different national backgrounds will also be further expanded.

Finally, a visualization analysis was conducted on the journals and literature sources related to CL in PE. It was found that the journals focusing on PE were the main sources of research on CL in this field. The top-ranked journal was *Journal of Teaching in Physical Education*, with 454 citations and a connection strength of 8,673. The second-ranked journal was *Physical Education and Sport Pedagogy*, with 264 citations and a connection strength of 6,097. Following these were *Sport, Education and Society*, *Quest*, and *European Physical Education*.

## 6 Conclusion and limitation

In this study, a systematic review and visual analysis of 169 documents on CL in PE from the WOS database was conducted. The specific status of CL in PE was examined using bibliometrics and following the specific processes of systematic review and meta-analysis, and the visual analysis software CiteSpace and VOSviewer were used to construct a knowledge map. The individual learning model has important research implications for the development of both learners’ and educators’ competencies in the process of changing to CL (Liu and Lipowski, 2021). It is important for the development of intrinsic motivation of learners, increasing classroom participation, and improving teachers’ ability to use flexible teaching methods. Furthermore, we observed that the application of CL in PE is primarily concentrated in primary and secondary education and teacher training, with a secondary focus on university-level education. In primary and secondary education, the emphasis is primarily on student motivation, teacher-student interaction, group dynamics, and social learning. In college, the focus shifts toward enhancing students’ physical skills, learning efficiency, and participation, particularly among female students. Concerning teacher training, there is a stronger inclination toward fostering teaching methods and technological proficiency among educators. The promotion of the overall development of learners (motor, cognitive, social and affective) and the teaching ability of educators, as well as the adaptation for different teaching environments and the integration of different teaching methods still need to be further improved (Evangelio et al., 2021). In the future, for the application of CL in PE, it is not only necessary to focus on combining with the current teaching techniques, but also to continue to pay attention to the development of learners’ and educators’ abilities.

This study also has some limitations, firstly, this study only describes the current dynamic trends of CL in PE from the perspective of systematic review, and does not provide a specific review through quantitative methods; secondly, the data cleaning process of the literature, in order to improve the quality of the articles reviewed, we removed the articles from conferences, posters, books, and other languages, and in the future, we should expand the scope of the literature data; Finally, the diversity of visualization and analysis software in bibliometrics, only two of them were used in this paper for the analysis, every research method has its limitations, and future studies will further conduct a specific examination and categorization of the effects of CL on educational outcomes to make the research information more clear in the future. Through this study, we hope that it can help learners and educators to provide a lot of useful and valuable information for the development and research of CL in PE.

## Author contributions

TZ: Writing – original draft, Writing – review & editing. HW: Visualization, Validation. DL: Writing – review & editing.
